# Therapeutic effects of Jing Si herbal tea for chronic obstructive pulmonary disease: a comprehensive investigation from clinical to basic research

**DOI:** 10.3389/fphar.2025.1631839

**Published:** 2025-07-21

**Authors:** Chou-Chin Lan, Po-Chun Hsieh, Kuo-Liang Huang, Mei-Chen Yang, Guan-Ting Liu, Chan-Yen Kuo, I.-Shiang Tzeng, Yao-Kuang Wu

**Affiliations:** ^1^ Division of Pulmonary Medicine, Taipei Tzu Chi Hospital, Buddhist Tzu Chi Medical Foundation, New Taipei, Taiwan; ^2^ School of Medicine, Tzu Chi University, Hualien, Taiwan; ^3^ Department of Chinese Medicine, Taipei Tzu Chi Hospital, Buddhist Tzu Chi Medical Foundation, New Taipei, Taiwan; ^4^ Department of Research, Taipei Tzu Chi Hospital, Buddhist Tzu Chi Medical Foundation, New Taipei, Taiwan

**Keywords:** chronic obstructive pulmonary disease, damage-associated molecular patterns, Jing Si herbal tea, inflammation, cytokines

## Abstract

**Background:**

Chronic obstructive pulmonary disease (COPD), a leading cause of global mortality, significantly impairs health-related quality of life (HRQL). COPD is characterized by airway inflammation and lung tissue damage. Jing Si herbal tea (JSHT) has anti-inflammatory effects but has not been explored for treating COPD. This study investigated the potential of JSHT as an adjuvant therapy for COPD.

**Methods:**

This study focused on patients with COPD in the exacerbation and stable phases. The control group received the standard treatment, and the JSHT group received the standard treatment plus JSHT. Both groups underwent HRQL assessments, blood tests, and cellular studies involving five different groups to assess the effect of JSHT on damage-associated molecular patterns (DAMPs) and inflammatory markers.

**Results:**

Among patients with exacerbations, the JSHT group showed significant improvements in HRQL, including reductions in cough, phlegm, chest tightness, breathlessness, sleep, and anxiety (all p < 0.05). Among patients with stable COPD, the JSHT group showed significant reductions in cough, phlegm, and breathlessness (all p < 0.05). Cellular studies on lipopolysaccharide (LPS)-stimulated A549 cells demonstrated that JSHT effectively reduced the release of DAMPs such as HMGB1, FPR1, and extracellular ATP, and decreased the expression of inflammatory markers including pMAPK, pJNK, NF-kB, and cCaspase 3, and pro-inflammatory cytokines like IL-1, IL-6, IL-8, and TNF-α post-LPS induction.

**Conclusion:**

JSHT improved the HRQL in patients with COPD, both in stable and exacerbated states. Cellular models demonstrated a reduction in DAMPs and inflammation, suggesting the potential of JSHT as a therapeutic agent for COPD through modulation of inflammatory responses.

## 1 Introduction

Chronic obstructive pulmonary disease (COPD), characterized by irreversible airflow limitation, is the third leading cause of death worldwide and a major contributor to chronic morbidity and healthcare burden globally (Venkatesan, 2025). Individuals with COPD typically suffer from progressive lung damage and airway obstruction (Jarab et al., 2023). The condition is associated with symptoms such as chronic cough, phlegm production, dyspnea, wheezing, fatigue, and reduced exercise tolerance, all of which significantly impair health-related quality of life (HRQL) (Jarab et al., 2023). Despite adherence to optimal medical therapies, many patients continue to experience substantial disease burden and exacerbations (Venkatesan, 2025).

Harmful agents such as cigarette smoking and respiratory infections are the primary risk factors for COPD (Kotlyarov, 2023). The pathogenesis of COPD is driven by chronic airway inflammation and epithelial damage (Kotlyarov, 2023). Persistent exposure to noxious agents leads to epithelial cell injury and the release of damage-associated molecular patterns (DAMPs), (Cristaldi et al., 2023), which trigger inflammatory cascades involving key cytokines such as interleukin (IL)-1, IL-6, IL-8, and tumor necrosis factor (TNF)-α (Kotlyarov, 2023). These cytokines further activate immune cells, perpetuating inflammation and tissue destruction (Kotlyarov, 2023). Consequently, interventions that interrupt DAMP-driven inflammatory cascades may represent novel adjunctive therapies for COPD.

Recent studies have highlighted the therapeutic potential of natural products as adjuvant treatments for COPD due to their anti-inflammatory properties. Several plant-derived metabolites have been shown to suppress the release of damage-associated molecular patterns (DAMPs), inhibit key inflammatory pathways such as NF-κB and MAPK, and reduce the production of pro-inflammatory cytokines, including IL-1β, IL-6, IL-8, and TNF-α in COPD-related models (Mao et al., 2020; Sun et al., 2021; Liu et al., 2022). For example, glycyrrhizin from Glycyrrhiza glabra and casticin from Chrysanthemum morifolium have demonstrated the ability to attenuate lung inflammation and oxidative stress (Sun et al., 2021). Polysaccharide extracts from Ophiopogon japonicus and Houttuynia cordata have also been reported to modulate airway inflammation by regulating MAPK/NF-κB signaling (Mao et al., 2020). These findings support the use of plant-based interventions to modulate chronic airway inflammation and improve clinical outcomes in COPD.

Jing Si Herbal Tea (JSHT) is a polyherbal formulation developed to modulate the immune response (Hsieh et al., 2022a). A recent study demonstrated that the addition of JSHT to standard COVID-19 therapy significantly reduced C-reactive protein levels and improved clinical outcomes, including lower rates of intubation, disease severity, and mortality (Hsieh et al., 2022a). These findings suggest the potential of JSHT as an immune-modulatory adjunct in inflammatory diseases (Hsieh et al., 2022a). However, its therapeutic role in COPD has not yet been explored.

In this study, we evaluated whether JSHT attenuates DAMP release and downstream pro-inflammatory cytokine expression in a cellular model of COPD. Our aim was to investigate the clinical benefits and potential mechanisms by which JSHT may modulate airway inflammation, providing a foundation for its future clinical application in COPD management.

## 2 Materials and methods

### 2.1 Clinical study and patient selection

This prospective, parallel-group study was conducted from 1 April 2022, to 31 July 2023, and included patients with acute exacerbation of COPD (COPDAE) and patients with stable COPD. Participants were randomly assigned in a 1:1 ratio to receive either JSHT or placebo using a computer-generated randomization sequence. Blinding was maintained throughout the study: participants, healthcare providers, and outcome assessors were all blinded to treatment allocation.

In the COPDAE group, patients in the control arm received standard treatment (intravenous corticosteroids, inhaled butanyl and ipratropium, and antibiotics if indicated) plus a placebo resembling JSHT. The JSHT group received the same standard treatment plus JSHT (one pack, three times daily for 1 week).

In the stable COPD group, both arms received standard inhaled therapy according to clinical guidelines. The JSHT group additionally received one pack of JSHT daily for 3 months, while the control group received a matched placebo.

HRQL assessments, pulmonary function tests (PFT) and laboratory tests were conducted at baseline and post-treatment. The study was approved by the Ethics Committee of Taipei Tzu Chi Hospital (IRB number 10-XD-132), and written informed consent was obtained from all participants.

### 2.2 Components of JSHT and placebo

JSHT is a traditional Chinese botanical drugs developed by the Buddhist Tzu Chi Medical Foundation and consists of the following botanical drugs: Houttuynia cordata Thunb. (Houttuyniae Herba, family Saururaceae, aerial parts, 14.18%), Perilla frutescens (L.) Britton (Perillae Folium, Lamiaceae, leaf, 7.09%), Glycyrrhiza glabra L. (Glycyrrhizae Radix et Rhizoma, Fabaceae, root, 7.09%), Artemisia argyi H. Lév. and Vaniot (Artemisiae Argyi Folium, Asteraceae, leaf, 21.28%), Anisomeles indica (L.) Kuntze (Anisomeles Herba, Lamiaceae, whole herb, 21.28%), Platycodon grandiflorus (Jacq.) A. DC. (Platycodonis Radix, Campanulaceae, root, 14.18%), Ophiopogon japonicus (Thunb.) Ker Gawl. (Ophiopogonis Radix, Asparagaceae, tuber, 14.18%), and Chrysanthemum morifolium Ramat. (Chrysanthemi Flos, Asteraceae, flower, 0.71%) (Hsieh et al., 2022a). The herbal formulation includes the following botanical ingredients and their used plant parts: Argyi Leaf (leaf), Kalabhangra (leaf and stem), Ophiopogonis Radix (tuberous root), Dokudami (whole plant), Balloon Flower (root), Glycyrrhizae Radix (root and rhizome), Perilla (leaf), and Chrysanthemi Flos (flower). The preparation was produced via aqueous extraction, filtration, and concentration, and packaged in GMP-certified 225 mL vacuum-sealed single-dose pouches (Hsieh et al., 2022a). The placebo used in this study was formulated with grass jelly, designed to closely resemble the JSHT preparation in taste, color, appearance, and packaging. This sensory matching was implemented to maintain effective blinding for both participants and investigators.

## 3 Preparation of JSHT

For *in vitro* use, the JSHT passed through a 0.22 μm syringe filter, and stored at −20°C until use.

### 3.1 Dose selection

We appreciate the reviewer’s request for clarification. The concentrations used in this study 0.5% v/v for JSHT was selected based on both our prior experimental experience and findings reported in our previous publication (Wei et al., 2024). The concentration of 100 μg/mL LPS was chosen based on prior studies demonstrating robust inflammatory activation in epithelial cell models without compromising cell viability (Hsieh et al., 2022b). Dose-response pilot experiments confirmed this concentration was sufficient to induce inflammatory cytokinase in our model system.

### 3.2 Laboratory tests

Blood tests including white blood cells (WBCs), percentages of different types of WBCs (neutrophils, lymphocytes, monocytes, eosinophils, basophils), hemoglobin (Hb), hematocrit (Hct), platelets (PLT), blood urea nitrogen (BUN), creatinine (Cr), uric acid (UA), liver enzymes (aspartate aminotransferase, alanine aminotransferase), electrolytes (sodium, potassium), C-reactive protein (CRP), and pro-brain natriuretic peptide (pPro-BNP).

### 3.3 HRQL

The Taiwan Society of Pulmonary and Critical Care Medicine provides a Chinese version of the COPD Assessment Test (CAT) (Huang et al., 2022). The CAT, consisting of eight items, assesses COPD symptoms such as cough, phlegm, chest tightness, breathlessness, limitations in daily activities, confidence in leaving the house, sleep disturbances, and energy levels. Each symptom is rated on a scale of 0–5, with a total score ranging from 0 to 40. A score of 10 or higher indicates a significant symptom burden (Huang et al., 2022). The Modified Medical Research Council (mMRC) scale, a 5-point grading system from 0 to 4, assesses dyspnea severity. A score of 0 indicates dyspnea only during intense exercise, while a score of 4 signifies breathlessness at rest (MJ Tsai et al., 2012). The 5-item Brief Symptom Rating Scale (BSRS-5) was used to assess psychological distress. The scale is a 5-point scale ranging from 0 (not at all) to 4 (extremely), with higher scores indicating more severe symptoms (Lin et al., 2023; Yang et al., 2024).

### 3.4 Pulmonary function tests

PFTs were performed using a calibrated spirometer (Medical Graphics Corp., St. Paul, MN, United States) in accordance with the guidelines of the American Thoracic Society (ATS) (Culver BH et al., 2017). The spirometric parameters measured included forced vital capacity (FVC), forced expiratory volume in one second (FEV_1_), the FEV_1_/FVC ratio, and maximal mid-expiratory flow (MMEF). All measurements were obtained in the pre-bronchodilator condition to evaluate baseline pulmonary function and the effects of treatment. All measurements were obtained under both pre-bronchodilator and post-bronchodilator conditions to assess baseline PFTs and treatment effects. As per the Global Initiative for Chronic Obstructive Lung Disease (GOLD) recommendations, post-bronchodilator values were used for the diagnosis and classification of airflow limitation severity (Agustí et al., 2023). Quality control procedures were rigorously followed, including daily calibration of the spirometer, regular maintenance checks, and verification of acceptable test reproducibility and performance criteria for each patient.

### 3.5 Cellular study for assessing the effects of JSHT on inflammation

In the cellular study conducted on A549 cells, five groups were analyzed to evaluate the effects of JSHT. The groups were: 1) Control group: did not receive treatment; 2) lipopolysaccharide (LPS) group: treated with 100 ug/mL LPS for 16 h to induce inflammation; 3) JSHT group: treated with 0.5 volume % JSHT for 12 h; 4) Pre-JSHT + LPS group: cells were pre-treated with 0.5 volume % JSHT for 1 h, then 100 ug/mL LPS treatment for 16 h; 5) Post-JSHT + LPS group: cells were exposed to 100 ug/mL LPS for 16 h followed by 0.5 volume % JSHT for 12 h. Measurements were performed for DAMPs such as high mobility group box 1 (HMGB1), formyl peptide receptor 1 (FPR1), and extracellular adenosine triphosphate (ATP); transcription factor nuclear factor kappa-light-chain-enhancer of activated B cells (NF-κB); phosphorylated mitogen-activated protein kinase (p-MAPK) and c-Jun N-terminal kinase (p-JNK); apoptotic marker cleaved Caspase 3 (cCaspase 3); and pro-inflammatory cytokines, including interleukin-1 (IL-1), interleukin-6 (IL-6), interleukin-8 (IL-8), and tumor necrosis factor-alpha (TNF-α).

### 3.6 Cell line authentication

The A549 human lung epithelial cell line used in this study was obtained from the American Type Culture Collection (ATCC, CCL-185). Cell line authentication was confirmed by short tandem repeat (STR) profiling, and cells were routinely screened for *mycoplasma* contamination using a PCR-based detection kit (MycoAlert™ *Mycoplasma* Detection Kit, Lonza). All experiments were conducted with cells within 20 passages after thawing.

### 3.7 Measurement of DAMPs

A549 cells (1 × 10^6^) were seeded in 6-well plates and incubated at 37°C with 5% CO_2_ for 24 h. Following exposure to five different experimental conditions, the culture medium was harvested, centrifuged at 300 × g for 10 min, and the supernatant was stored at −80°C until analysis. The levels of DAMPs, including HMGB1, FPR1, and extracellular ATP, were measured using commercially available ELISA kits. HMGB1 and ATP were quantified using kits from Abclonal (catalog numbers RK06736 and RK04252, respectively), and FPR1 was measured using a kit from Mybiosource (catalog number MBS9336637). The detection limits were 0.1 ng/mL for HMGB1, 0.35 ng/mL for ATP, and 1.0 ng/mL for FPR1.

### 3.8 Measurement of cCaspase-3, NF-kB, p-MAPK, and p-JNK

After treatment, A549 cells were lysed in cold RIPA buffer supplemented with protease and phosphatase inhibitors. Protein concentrations were determined using a BCA protein assay kit. Equal amounts of total protein were separated by electrophoresis using 4%–12% SDS-PAGE gels at 140 V for 1 h, followed by transfer onto PVDF membranes at 200 mA for 2 hours. Membranes were blocked with TOOLSpeed blocking reagent for 1 h at room temperature and incubated overnight at 4°C with primary antibodies against cleaved caspase-3 (Cell Signaling, #9961, 1:1000 dilution, validated in PMID: 39878157), NF-κB (Abclonal, A2547, 1:1000, PMID: 26202983), phosphorylated MAPK (p-MAPK; Abclonal, AP0526, 1:1000, PMID: 31281493), and phosphorylated JNK (p-JNK; Abclonal, AP0631, 1:1000, PMID: 31614673). GAPDH (Abclonal, AC027, 1:1000, PMID: 29727616) was used as the loading control.

After washing, membranes were incubated with appropriate HRP-conjugated secondary antibodies for 1 hour at room temperature. Signals were detected using enhanced chemiluminescence (ECL) reagents and visualized with the Bio-Rad ChemiDoc XRS+ imaging system. Band intensities were quantified using ImageJ software. The expression level of each target protein was normalized to GAPDH and presented as the ratio of target to GAPDH to account for loading variability.

### 3.9 Cytokines ELISA

A549 cells were cultured in a 6-well plate at 37°C for 24 h. After treatment under five experimental conditions, the culture medium was collected and stored at −80°C. Inflammatory cytokines including interleukin-1β (IL-1β), IL-6, IL-8, and tumor necrosis factor-α (TNF-α) were quantified using ELISA kits from Abclonal (catalog numbers RK00001, RK00004, RK00011, and RK00030, respectively). The detection limits for these cytokines were 3.9 pg/mL for IL-1β, 0.7 pg/mL for IL-6, 1.7 pg/mL for IL-8, and 6.9 pg/mL for TNF-α. All ELISA procedures were conducted according to the manufacturers’ instructions. Each sample was assayed in triplicate to ensure accuracy and reproducibility. Absorbance readings were obtained using an Infinite 200 PRO microplate reader (Tecan, Männedorf, Switzerland).

### 3.10 Statistical analysis

Data are presented as mean ± standard deviation (SD). Statistical analyses were conducted using SPSS (version 24.0). For the clinical study, paired t-tests compared baseline and post-treatment outcomes within each group. In the cellular study, ANOVA assessed differences among the five groups (control, JSHT, LPS, pre-JSHT, post-JSHT), with *post hoc* comparisons using the least significant difference method. Statistical significance was set at p < 0.05.

## 4 Results

### 4.1 Effects of JSHT in patients with COPD acute exacerbation (COPDAE)

There were 4 and 5 patients with COPDAE in the control and JSHT groups, respectively. The baseline characteristics of the patients with COPDAE are shown in Table 1. Age, sex, smoking status, BH, BW, BMI, and lung function test results were not significantly different between the two groups (all p > 0.05).

**TABLE 1:** Baseline characteristics of patients with COPDAE.

Variable	Control (n = 4)	JSHT (n = 5)	p
Age	67.8 ± 6.7	69.0 ± 7.6	0.801
Sex (M/F)	4/0	5/0	1.000
Smoking (No/current/ex)	0/1/3	0/1/4	0.858
BH (cm)	162.3 ± 2.1	164.6 ± 4.5	0.371
BW (Kg)	63.9 ± 8.9	74.0 ± 15.1	0.359
BMI (kg/m^2^)	24.3 ± 3.9	27.2 ± 4.8	0.280
FEV1/FVC (%)	45.7 ± 11.7	55.3 ± 15.3	0.129
FVC (L)	2.5 ± 0.4	3.2 ± 0.8	0.129
FVC (%)	86.3 ± 15.0	102.6 ± 29.1	0.412
FEV1 (L/sec)	1.1 ± 0.4	1.7 ± 0.5	0.067
FEV1 (%)	52.0 ± 13.1	68.0 ± 18.1	0.215
Medications			
Parenteral antibiotics	4/4	5/5	
Intravascular steroid	4/4	5/5	
Inhaled ipratropium	4/4	5/5	
Inhaled butanyl	4/4	5/5	

Abbreviations: COPD: chronic obstructive pulmonary disease; JSHT: jing si herbal tea; BH: body height; BW: body weight; BMI: body mass index; FEV: forced expiratory volume in one second; FVC: forced vital capacity.

The HRQL values are listed in Table 2. In the control group, improvements were observed in the CAT total score, chest tightness, breathlessness, limitation of activities, dyspnea, mMRC score, and sleep difficulties (p < 0.05). In the JSHT group, significant improvements were observed in more aspects, including the CAT total score, cough, phlegm, chest tightness, breathlessness, limitation of activities, confidence in leaving home, mMRC dyspnea, sleep difficulties, and anxiety (p < 0.05).

**TABLE 2:** Health-related quality of life of patients with COPDAE.

Variable	Control (n = 4)		P	JSHT (n = 5)		P
	baseline	treated		baseline	treated	
CAT total score	23.8 ± 9.2	16.0 ± 7.9	0.002	27.2 ± 5.8	15.2 ± 4.8	0.001
Cough	2.5 ± 2.1	1.8 ± 1.5	0.215	3.4 ± 1.8	2.0 ± 1.6	0.033
Phlegm	1.8 ± 1.7	1.5 ± 1.3	0.391	3.4 ± 1.5	1.6 ± 1.3	0.009
Chest tightness	3.8 ± 1.3	2.5 ± 1.3	0.015	4.0 ± 1.2	1.4 ± 1.3	0.003
Breathlessness	4.3 ± 0.5	3.0 ± 0.8	0.015	3.6 ± 1.7	2.4 ± 0.9	0.033
Limitation of activities	3.3 ± 1.0	1.8 ± 0.5	0.014	3.0 ± 1.9	1.8 ± 1.1	0.033
Confidence leaving home	2.3 ± 1.7	1.3 ± 1.0	0.092	3.0 ± 1.6	1.6 ± 1.1	0.025
Sleep	3.3 ± 2.2	2.5 ± 2.1	0.215	3.6 ± 1.7	2.6 ± 1.8	0.089
Energy	2.8 ± 1.0	1.8 ± 1.0	0.092	3.2 ± 0.8	1.8 ± 0.8	0.080
mMRC	3.5 ± 0.6	2.3 ± 0.5	0.015	3.4 ± 1.0	2.2 ± 0.4	0.033
Sleep difficulties	4 ± 0	2.3 ± 1.0	0.035	3.0 ± 1.2	1.4 ± 1.1	0.035
Feeling tense	1.0 ± 1.2	0.5 ± 0.6	0.182	1.2 ± 0.8	0.2 ± 0.4	0.034
Easily angered	0.5 ± 0.6	0 ± 0	0.182	0.8 ± 1.1	0.2 ± 0.4	0.208
Depression	1.5 ± 1.7	0.5 ± 1.0	0.252	1.6 ± 1.5	0.2 ± 0.4	0.080
feeling inferior to others	1.3 ± 1.5	0.5 ± 1.0	0.215	0.8 ± 0.8	0.0 ± 0.0	0.099
Suicidal thoughts	0.0 ± 0.0	0.0 ± 0.0	1.000	0.2 ± 0.4	0.0 ± 0.0	0.374
BRRS-total score	8.3 ± 4.9	3.8 ± 2.2	0.131	7.6 ± 5.1	2.0 ± 2.0	0.041

Abbreviations: COPD: chronic obstructive pulmonary disease; JSHT: jing si herbal tea; CAT: COPD, assessment test; mMRC: modified medical research council; BRRS: Borg rating of perceived exertion-rating scale.

Laboratory data of patients with COPDAE are shown in Table 3. In both groups, most parameters, including blood cell count, hemoglobin level, renal function, and liver function, showed no significant differences after treatment (p > 0.05). However, a significant decrease in potassium levels was observed in the control group (p = 0.017), which was not evident in the JSHT group (p = 0.098). The JSHT group showed a reduction in CRP levels (from 5.7 ± 8.5 to 1.1 ± 1.6), but this change did not reach statistical significance (p > 0.05).

**TABLE 3:** Laboratory data of patients with COPDAE.

Variable	Control	Control	P	JSHT	JSHT	P
	baseline	treated		baseline	treated	
WBC (x10^3/uL)	11,992 ± 2,241	11,697 ± 2,428	0.817	10,428 ± 4,073	9,712 ± 2,730	0.564
Neutrophils (%)	70.7 ± 19.8	71.0 ± 12.8	0.970	66.9 ± 18.9	73.7 ± 8.6	0.319
Lymphocyte (%)	18.9 ± 14.8	21.8 ± 11.9	0.663	23.0 ± 16.9	16.9 ± 8.7	0.225
Monocyte (%)	3.7 ± 1.8	5.7 ± 0.2	0.108	6.8 ± 2.3	7.0 ± 2.2	0.826
Eosinophil (%)	6.1 ± 8.4	1.2 ± 1.1	0.345	2.8 ± 3.4	2.1 ± 1.7	0.749
Basophil (%)	0.4 ± 0.3	0.3 ± 0.2	0.670	0.5 ± 0.2	0.2 ± 0.2	0.034
Hb (g/dL)	14.6 ± 0.9	13.9 ± 1.3	0.185	16.8 ± 1.6	15.3 ± 1.6	0.128
Hct (%)	45.4 ± 2.0	42.6 ± 4.2	0.167	50.9 ± 4.5	46.4 ± 5.2	0.102
PLT (*10^3/uL)	260 ± 53	247 ± 48	0.526	204 ± 38	174 ± 85	0.402
BUN (mg/dL)	16.0 ± 8.7	18.0 ± 4.1	0.744	21.4 ± 9.2	27.0 ± 11.6	0.276
Cr (mg/dL)	0.9 ± 0.1	0.8 ± 0.1	0.130	1.3 ± 0.3	1.2 ± 0.4	0.304
UA (mg/dL)	6.0 ± 1.2	6.1 ± 0.7	0.699	6.6 ± 3.0	6.4 ± 3.0	0.730
AST (U/L)	20.1 ± 8.5	16.5 ± 9.3	0.274	24.4 ± 14.0	22.0 ± 13.3	0676
ALT (U/L)	22.5 ± 20.9	24.8 ± 16.3	0.662	21.0 ± 19.1	25.0 ± 13.0	0.334
Na (mmol/L)	137.8 ± 1.3	139.3 ± 1.7	0.297	135.8 ± 6.5	136.4 ± 3.9	0.666
K (mmol/L)	4.3 ± 0.3	3.5 ± 0.7	0.017	4.1 ± 0.2	3.8 ± 0.3	0.098
CRP (mg/dL)	1.7 ± 1.7	0.9 ± 1.1	0.077	5.7 ± 8.5	1.1 ± 1.6	0.259
Pro-BNP (pg/mL)	177.3 ± 128.9	167.5 ± 146.1	0.595	901.5 ± 398.1	901.0 ± 181.0	0.999

Abbreviations: COPD: chronic obstructive pulmonary disease; JSHT: jing si herbal tea; WBC: white blood cells; Hb: hemoglobin; Hct: hematocrit; PLT: platelets; BUN: blood urea nitrogen; Cr: creatinine; UA: uric acid; AST: aspartate aminotransferase; ALT: alanine aminotransferase; Na: sodium; K: potassium; CRP: C-reactive protein; Pro-BNP: pro-brain natriuretic peptide.

### 4.2 Effects of JSHT in patients with stable COPD

There were 8 and 9 patients with stable COPD in the control and JSHT groups, respectively. The baseline characteristics of the patients with stable COPD are shown in Table 4. Age, sex, smoking status, BH, BW, BMI, and lung function were not significantly different between the two groups (all p > 0.05).

**TABLE 4:** Baseline characteristics of patients with stable COPD.

Variable	Control (n = 8)	JSHT (n = 9)	p
Age	67.4 ± 9.1	70.2 ± 6.5	0.465
Sex (M/F)	8/0	8/1	1.000
Smoking (No/current/ex)	0/3/5	1/3/5	0.515
BH (cm)	163.4 ± 6.6	162.2 ± 4.7	0.681
BW (Kg)	62.8 ± 12.3	66.9 ± 14.3	0.541
BMI (kg/m^2^)	23.6 ± 4.4	25.3 ± 4.4	0.435
FEV1/FVC (%)	57.6 ± 20.2	51.7 ± 12.3	0.489
FVC (L)	2.6 ± 0.8	2.9 ± 0.9	0.582
FVC (%)	86.4 ± 13.8	94.4 ± 27.7	0.636
FEV1 (L/sec)	1.5 ± 0.6	1.5 ± 0.6	0.982
FEV1 (%)	60.4 ± 23.7	61.2 ± 19.2	0.914
Medication			
LAMA + LABA + ICS	8/8	7/9	
LAMA + LABA	0/8	2/9	

Abbreviations: COPD: chronic obstructive pulmonary disease; JSHT: jing si herbal tea; BH: body height; BW: body weight; BMI: body mass index; FEV: forced expiratory volume in one second; FVC: forced vital capacity; LAMA: long-acting muscarinic antagonist; LABA: long-acting beta agonist; ICS: inhaled corticosteroid.

The HRQL scores of patients with stable COPD are shown in Table 5. Significant reductions in breathlessness and mMRC scores were observed in the control group (p < 0.05). The total CAT score and cough, phlegm, chest tightness, and other symptoms also decreased; however, these changes were not statistically significant (p > 0.05). In the JSHT group, there were significant decreases in the total CAT score, cough, phlegm, breathlessness, and mMRC score (all p < 0.05). However, the BSRS scores were not significantly different post-treatment in either the control or JSHT groups (all p > 0.05).

**TABLE 5:** Health-related quality of life of patients with stable COPD.

Variable	Control (n = 8)		P	JSHT (n = 9)		P
	baseline	treated		baseline	treated	
CAT total score	18.7 ± 4.2	15.5 ± 2.7	0.052	17.9 ± 4.2	12.9 ± 4.4	0.003
Cough	2.9 ± 0.4	2.5 ± 0.5	0.080	2.8 ± 1.4	1.4 ± 1.2	0.029
Phlegm	2.6 ± 1.1	2.4 ± 0.9	0.626	2.7 ± 1.5	1.6 ± 1.0	0.007
Chest tightness	2.6 ± 1.1	2.3 ± 0.9	0.442	1.8 ± 1.4	1.2 ± 1.1	0.302
Breathlessness	3.3 ± 0.7	2.4 ± 0.7	0.006	3.1 ± 0.9	1.8 ± 1.0	<0.001
Limitation of activities	1.8 ± 0.7	1.8 ± 0.7	1.000	1.8 ± 1.0	1.4 ± 1.3	0.545
Confidence leaving home	1.0 ± 0.9	0.6 ± 0.5	0.285	1.4 ± 1.0	1.3 ± 1.3	0.799
Sleep	2.4 ± 1.7	2.0 ± 1.7	0.402	2.8 ± 1.6	2.4 ± 1.8	0.500
Energy	1.8 ± 0.5	1.1 ± 0.8	0.095	1.6 ± 0.7	1.8 ± 0.8	0.347
mMRC	2.2 ± 1.4	1.4 ± 0.5	0.021	2.3 ± 0.5	1.3 ± 0.5	0.003
Sleep difficulties	2.4 ± 1.3	1.9 ± 1.1	0.316	2.2 ± 1.1	1.7 ± 1.4	0.214
Feeling tense	0.5 ± 0.5	0.4 ± 0.5	0.598	0.6 ± 1.0	0.6 ± 0.5	1.000
Easily angered	0.0 ± 0.0	0.1 ± 0.4	0.351	0.7 ± 0.9	0.3 ± 0.5	0.195
Depression	0.3 ± 0.7	0.4 ± 0.5	0.598	0.4 ± 0.8	0.1 ± 0.3	0.195
feeling inferior to others	0.4 ± 0.7	0.0 ± 0.0	0.197	0.2 ± 0.7	0.1 ± 0.3	0.347
Suicidal thoughts	0.0 ± 0.0	0.0 ± 0.0		0.1 ± 0.3	0.0 ± 0.0	0.347
BRRS total score	3.5 ± 1.8	2.8 ± 2.1	0.390	4.2 ± 3.6	2.8 ± 2.1	0.096

Abbreviations: COPD: chronic obstructive pulmonary disease; JSHT: jing si herbal tea; CAT: COPD, assessment test; mMRC: modified medical research council; BRRS: Borg rating of perceived exertion-rating scale.

The laboratory data of the patients with stable COPD are shown in Table 6. In both groups, most parameters, including blood cell count, Hb, electrolytes, renal function, and liver function, showed no significant differences after treatment (p > 0.05). In the JSHT group, percentage of basophils showed a significant decrease from 0.5% ± 0.3% to 0.3% ± 2.0% (p = 0.036).

**TABLE 6:** Laboratory data of patients with stable COPD.

Variable	Control (n = 8)		P	JSHT (n = 9)		P
	baseline	treated		baseline	treated	
WBC (x10^3/uL)	10,260.0 ± 3,368.8	9,497.5 ± 2,219.3	0.363	9,063.3 ± 2,267.5	7,943.3 ± 1830.2	0.159
Neutrophils (%)	69.3 ± 9.0	64.7 ± 12.9	0.223	72.6 ± 7.9	64.9 ± 13.2	0.085
Lymphocyte (%)	21.9 ± 8.5	22.7 ± 8.6	0.633	17.9 ± 7.6	25.2 ± 13.7	0.102
Monocyte (%)	6.2 ± 1.5	6.9 ± 1.6	0.117	6.5 ± 2.0	6.3 ± 1.5	0.712
Eosinophil (%)	2.2 ± 1.7	5.0 ± 5.8	0.213	2.6 ± 1.7	2.4 ± 2.0	0.762
Basophil (%)	0.5 ± 0.4	0.7 ± 0.4	0.527	0.5 ± 0.3	0.3 ± 2.0	0.036
Hb (g/dL)	14.4 ± 2.0	14.4 ± 1.9	1.000	14.6 ± 1.7	15.3 ± 2.0	0.292
Hct (%)	44.3 ± 5.1	44.2 ± 4.9	0.990	44.3 ± 5.0	46.2 ± 5.4	0.274
PLT (*10^3/uL)	230.1 ± 41.2	239.0 ± 57.2	0.621	205.0 ± 33.7	64.2 ± 21.4	0.484
BUN (mg/dL)	17.6 ± 4.5	18.0 ± 4.5	0.781	23.0 ± 9.8	19.2 ± 10.2	0.137
Cr (mg/dL)	1.0 ± 0.3	1.0 ± 0.2	0.289	1.0 ± 0.4	1.0 ± 0.4	0.869
UA (mg/dL)	5.7 ± 1.1	6.1 ± 1.6	0.083	6.9 ± 2.3	6.7 ± 1.8	0.553
AST (U/L)	18.1 ± 6.7	23.0 ± 15.8	0.255	21.0 ± 10.1	18.8 ± 3.4	0.575
ALT (U/L)	19.3 ± 12.8	24.4 ± 25.4	0.340	21.6 ± 11.2	16.5 ± 4.1	0.183
Bilirubin	0.6 ± 0.2	1.3 ± 2.0	0.360	0.7 ± 0.3	0.6 ± 0.9	0.333
Na (mmol/L)	139.1 ± 3.3	137.4 ± 2.5	0.133	137.9 ± 3.3	139.0 ± 2.7	0.247
K (mmol/L)	3.9 ± 0.6	4.1 ± 0.5	0.210	3.9 ± 0.4	4.0 ± 0.4	0.601
CRP (mg/dL)	1.2 ± 1.7	1.0 ± 1.6	0.783	0.8 ± 1.2	0.3 ± 0.4	0.999
Pro-BNP (pg/mL)	184.4 ± 168.1	113.4 ± 76.1	0.280	300.9 ± 379.2	213.4 ± 198.2	0.370
D-dimer	583.6 ± 731.6	621.9 ± 638.0	0.456	776.6 ± 653.8	777.2 ± 1104.4	0.999

Abbreviations: COPD: chronic obstructive pulmonary disease; JSHT: jing si herbal tea; WBC: white blood cells; Hb: hemoglobin; Hct: hematocrit; PLT: platelets; BUN: blood urea nitrogen; Cr: creatinine; UA: uric acid; AST: aspartate aminotransferase; ALT: alanine aminotransferase; Na: sodium; K: potassium; CRP: C-reactive protein; Pro-BNP: pro-brain natriuretic peptide.

The PFT results of patients with stable COPD are shown in Table 7. None of the PFT parameters showed significant differences after treatment in both groups (p > 0.05).

**TABLE 7:** Pulmonary function test of patients with stable COPD.

Variable	Control (n = 8)		P	JSHT (n = 9)		P
	baseline	treated		baseline	treated	
FEV1/FVC	57.6 ± 20.2	51.0 ± 14.6	0.169	51.7 ± 12.3	51.8 ± 13.3	0.938
FVC (L)	2.6 ± 0.8	2.9 ± 0.8	0.135	2.9 ± 0.9	2.9 ± 0.9	0.968
FVC (%)	89.4 ± 13.8	95.7 ± 15.0	0.185	94.4 ± 27.7	96.7 ± 24.7	0.562
FEV1 (L/min)	1.5 ± 0.5	1.5 ± 0.6	0.906	1.5 ± 0.6	1.5 ± 0.6	0.848
FEV1 (%)	62.4 ± 23.7	61.5 ± 19.9	0.835	61.2 ± 19.2	63.0 ± 20.9	0.617
MMEF	0.8 ± 0.4	0.7 ± 0.3	0.113	0.7 ± 0.3	0.8 ± 0.4	0.783
MMEF (%)	33.5 ± 19.1	27.4 ± 13.4	0.167	29.8 ± 11.6	30.4 ± 15.5	0.859

Abbreviations: COPD: chronic obstructive pulmonary disease; JSHT: jing si herbal tea; FEV: forced expiratory volume in one second; FVC: forced vital capacity; MMEF , maximum mid-expiratory flow.

### 4.3 Cellular investigation of the impact of JSHT on LPS-Stimulated A549 cells

**FIGURE 1:**
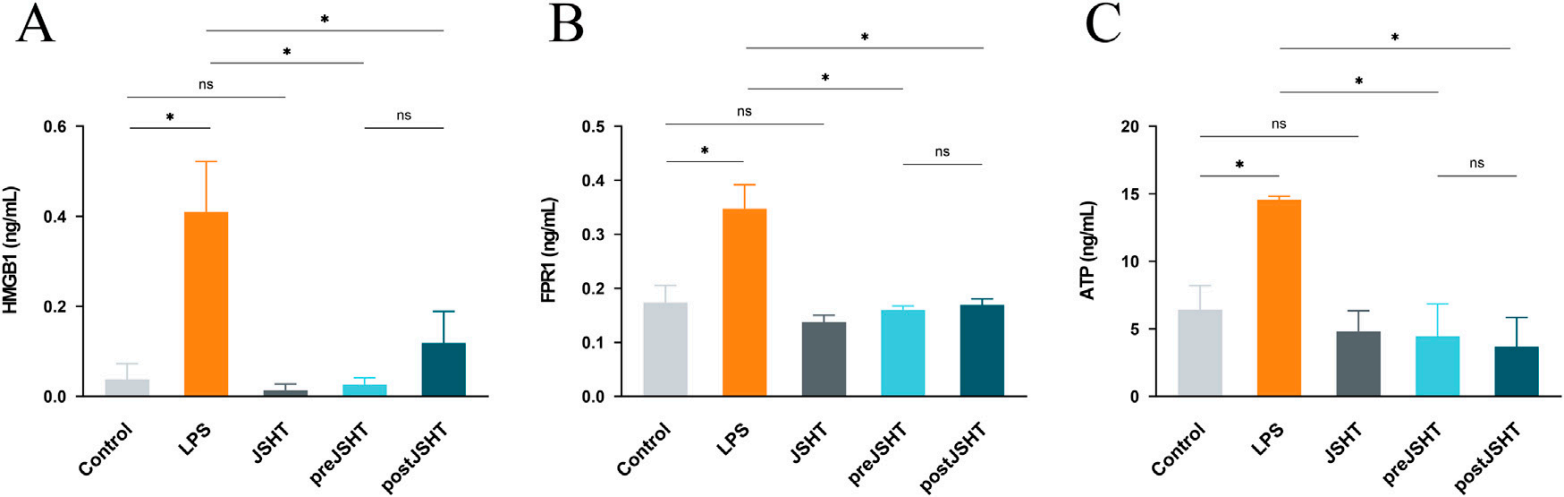
JSHT attenuates LPS-induced elevation in damage-associated molecular patterns (DAMPs) levels in A549 cells. ELISA results showing HMGB1 **(A)**, FPR1 **(B)**, and ATP **(C)** levels in A549 culture media after LPS exposure with or without JSHT treatment. LPS significantly increased these markers versus control (p < 0.05), while both pre- and post-JSHT groups significantly reduced them compared with LPS (p < 0.05). No significant differences were observed between pre- and post-JSHT groups (p > 0.05). *p < 0.05, NS = not significant. Abbreviations: HMGB1: high mobility group box 1; FPR1: formyl peptide receptor 1; ATP: adenosine triphosphate; LPS: lipopolysaccharide; JSHT: Jing Si Herbal Tea; pre-JSHT: Treatment with JSHT before LPS; post-JSHT: Treatment with JSHT after LPS.

The effects of JSHT on the attenuation of the LPS-induced release of DAMPs such as HMGB1, FPR1, and extracellular ATP in A549 cells are shown in Figure 1. Compared with the control group, LPS significantly increased HMGB1 (Figure 1A), FPR1 (Figure 1B), and ATP levels (Figure 1C) (all p < 0.05). Both the pre-JSHT and post-JSHT groups demonstrated significantly reduced levels of HMGB1, FPR1, and ATP compared with the LPS group (all p < 0.05). There were no significant differences between the pre-JSHT and post-JSHT groups (p > 0.05).

**FIGURE 2:**
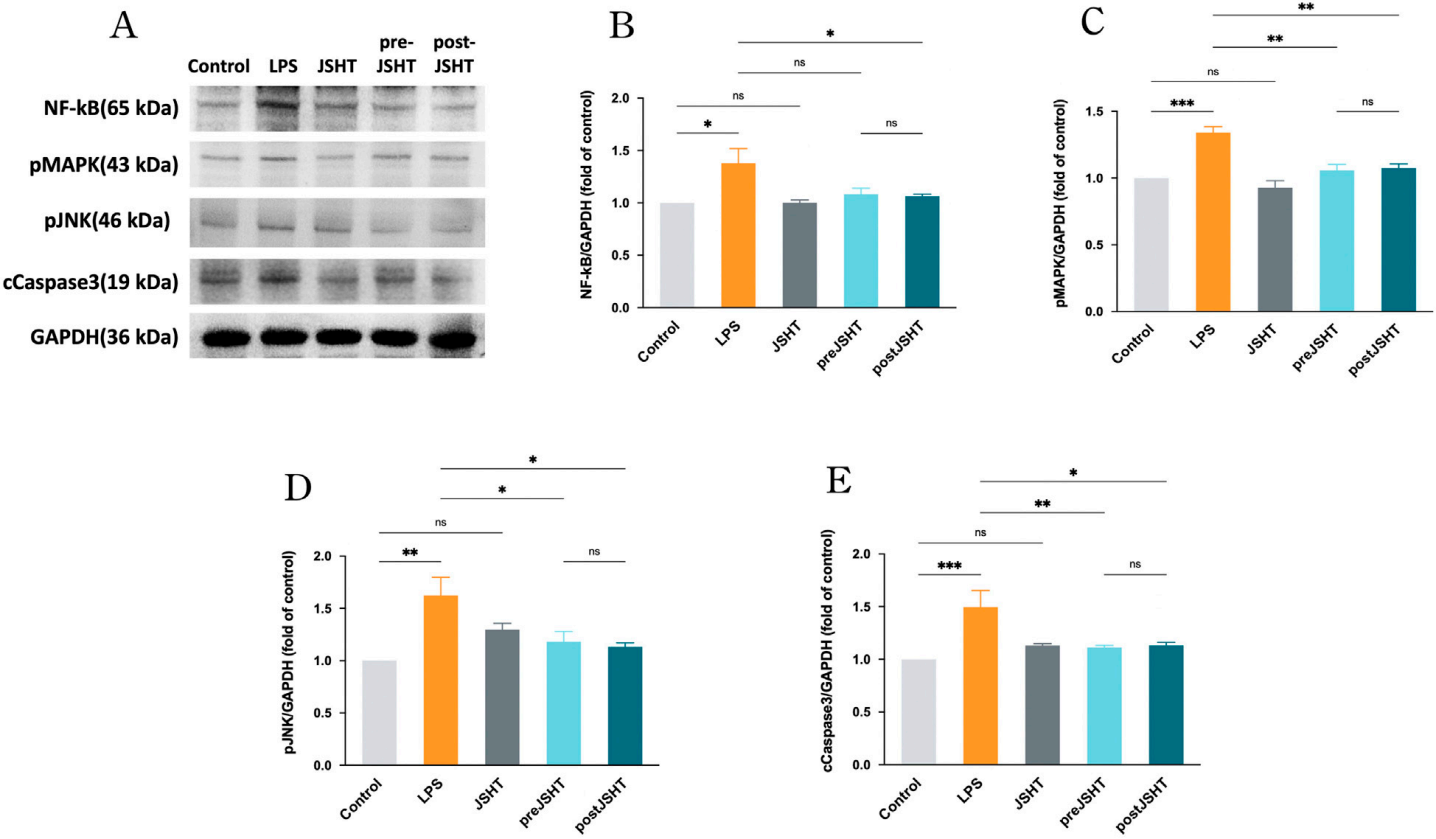
JSHT attenuated LPS-induced expression of NF-kB, pMAPK, pJNK, and cCaspase 3 in A549 cells. Western blot results showing **(A)** representative blots and quantified levels of **(B)** NF-κB, **(C)** pMAPK, **(D)** pJNK, and **(E)** cleaved caspase-3 in A549 cells after LPS exposure with or without JSHT treatment. LPS significantly increased all markers versus control (p < 0.05). Pre-JSHT reduced pMAPK, pJNK, and cleaved caspase-3, but not NF-κB, while post-JSHT reduced all markers compared with LPS (all p < 0.05). No significant differences were noted between pre-JSHT and post-JSHT groups (p > 0.05). *p < 0.05, NS = not significant. Abbreviations: NF-kB: nuclear factor kappa-light-chain-enhancer of activated B cells; pMAPK: phosphorylated mitogen-activated protein kinase; pJNK: phosphorylated c-jun N-terminal kinase; cCaspase3: cleaved caspase 3; GAPDH: glyceraldehyde 3-phosphate dehydrogenase; LPS: lipopolysaccharide; JSHT: Jing Si Herbal Tea; pre-JSHT: Treatment with JSHT before LPS; post-JSHT: Treatment with JSHT after LPS.

Figure 2 represents the Western blot results showing (Figure 2A) representative blots and quantified levels of NF-kB (Figure 2B), pMAPK (Figure 2C), pJNK (Figure 2D) and cCaspase 3 (Figure 2E) in A549 cells after LPS exposure with or without JSHT treatment. LPS significantly increased the expression of NF-κB, pMAPK, pJNK, and cleaved caspase-3 compared with the control group (all p < 0.05). The pre-JSHT group showed reduced levels of pMAPK, pJNK, and cleaved caspase-3 compared with the LPS group (all p < 0.05), but no significant difference in NF-κB (p > 0.05). The post-JSHT group showed reduced levels of NF-κB, pMAPK, pJNK, and cleaved caspase-3 compared with the LPS group (all p < 0.05). No significant differences were observed between the pre-JSHT and post-JSHT groups across all markers (p > 0.05).

**FIGURE 3:**
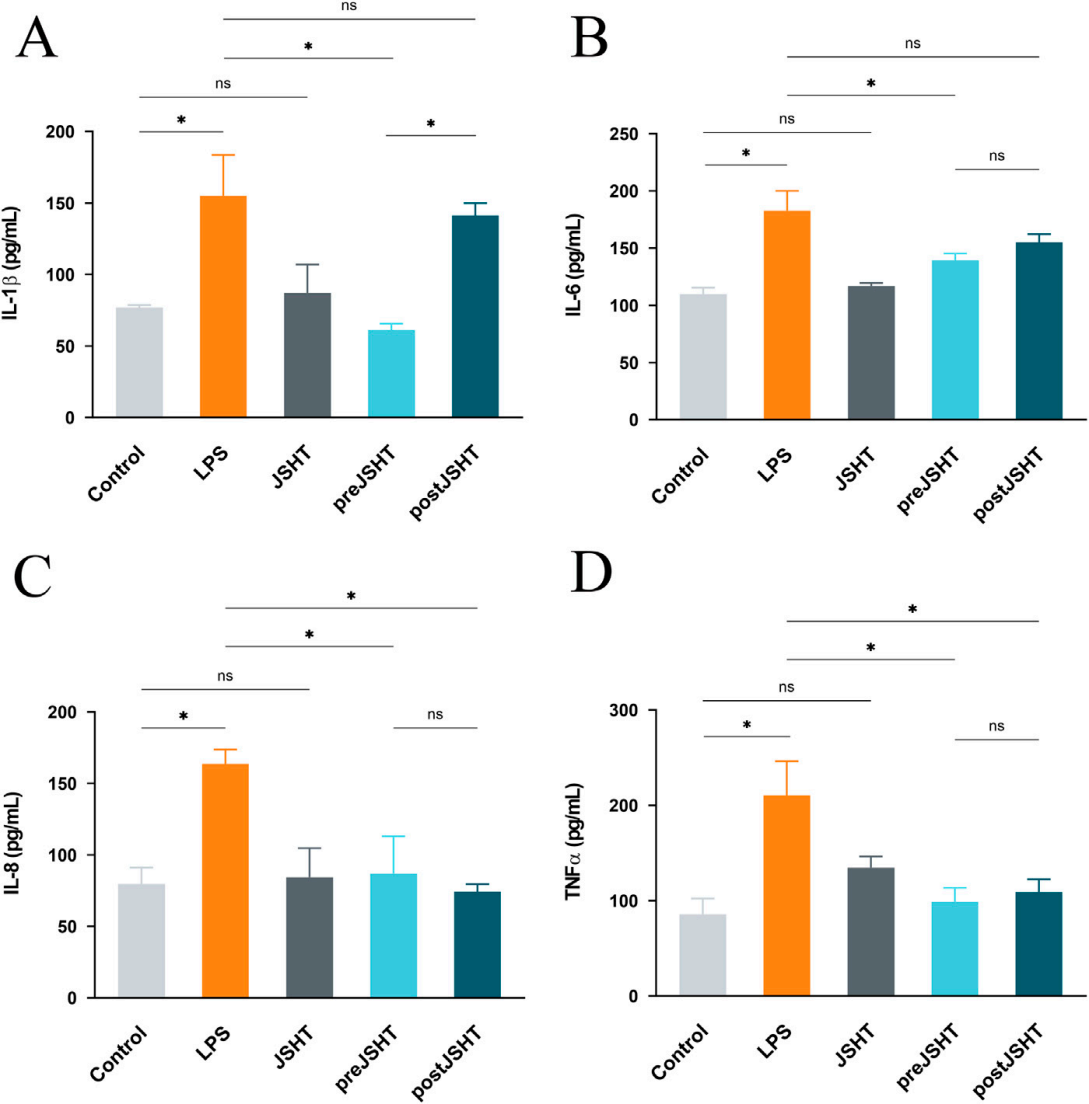
JSHT attenuated LPS-induced expression of pro-inflammatory cytokine levels in A549 cells ELISA results showing IL-1β **(A)**, IL-6 **(B)**, IL-8 **(C)**, and TNF-α **(D)** levels in A549 culture media after LPS exposure with or without JSHT treatment. LPS significantly increased all cytokines versus control (p < 0.05). Pre-JSHT reduced IL-1β, IL-6, IL-8, and TNF-α (p < 0.05), while post-JSHT reduced IL-8 and TNF-α (p < 0.05) but not IL-1β or IL-6 (p > 0.05). IL-1β was higher in post-JSHT than pre-JSHT (p < 0.05); no other significant differences were found between pre-JSHT and post-JSHT groups (p > 0.05). *p < 0.05, NS = not significant. IL-1: interleukin-1; IL-6: interleukin-6; IL-8: interleukin-8; TNF-α: tumor necrosis factor-alpha; LPS: lipopolysaccharide; JSHT: Jing Si Herbal Tea; pre-JSHT: Treatment with JSHT before LPS; post-JSHT: Treatment with JSHT after LPS.

LPS significantly increased levels of IL-1 (Figure 3A), IL-6 (Figure 3B), IL-8 (Figure 3C) and TNF-α (Figure 3D) compared with the control group (all p < 0.05). The pre-JSHT group significantly reduced IL-1β, IL-6, IL-8, and TNF-α levels compared with the LPS group (all p < 0.05). The post-JSHT group reduced IL-8 and TNF-α levels (p < 0.05), but showed no significant differences in IL-1β and IL-6 compared with the LPS group (p > 0.05). IL-1β levels were higher in the post-JSHT group than in the pre-JSHT group (p < 0.05), while no significant differences were observed between the pre-JSHT and post-JSHT groups for IL-6, IL-8, and TNF-α levels (p > 0.05).

## 5 Discussion

This study presents the clinical effects of JSHT on patients with COPDAE and stable COPD, as well as its cellular mechanisms in lung epithelial cells. The JSHT group exhibited broader improvements, including reductions in chest tightness, dyspnea, activity limitations, sleep difficulties, coughing, and phlegm; increased confidence in leaving home; and reduced anxiety in patients with COPDAE. In the stable COPD cohort, the JSHT group showed significant improvements in cough, phlegm, and dyspnea.

Cellular studies showed the mechanism of action of JSHT in COPD. LPS induces the release of DAMPs such as HMGB1, FPR1, and extracellular ATP in A549 cells. It also activated downstream inflammatory pathways such as NF-κB, MAPK, and cCaspase 3, along with pro-inflammatory cytokines including IL-1, IL-6, IL-8, and TNF-α, which are crucial to COPD pathogenesis. However, JSHT attenuates the LPS-induced release of DAMPs, pMAPK, pJNK, NF-κB, and cCaspase 3. This leads to a reduction in the levels of pro-inflammatory cytokines, highlighting the therapeutic potential of JSHT for the management of COPD.

The levels of inflammatory cytokines were markedly decreased following JSHT treatment, suggesting its potential anti-inflammatory effects. These cytokines also hold significant clinical relevance (Singh et al., 2018). In a previous clinical study, the serum levels of key inflammatory cytokines were found to be significantly elevated in patients with COPD compared to healthy controls (Singh et al., 2018). Specifically, the serum TNF-α level in COPD patients was 46.12 ± 10.83 pg/mL, significantly higher than the 22.47 ± 7.18 pg/mL observed in healthy individuals. IL-6 levels were also elevated in COPD patients at 33.78 ± 9.46 pg/mL, compared to 15.16 ± 4.72 pg/mL in controls. Similarly, IL-1β levels reached 23.34 ± 7.11 pg/mL in COPD patients, versus 10.65 ± 3.08 pg/mL in the control group (Singh et al., 2018). These cytokines not only differentiated COPD patients from healthy individuals but also demonstrated a progressive increase with advancing disease severity, as defined by GOLD stages, indicating a strong association between systemic inflammation and COPD progression (Singh et al., 2018). In our *in vitro* model, JSHT significantly suppressed IL-6 and TNF-α expression by over 40% relative to LPS-stimulated controls, reflecting a magnitude of inhibition that aligns with these clinically meaningful thresholds. Although direct extrapolation from cell culture to systemic effects in patients must be made cautiously, these results support the potential of JSHT to reduce inflammation to a degree that may translate into symptomatic and functional benefits in COPD management.

It is important to discuss the anti-inflammatory effects of JSHT. *Anisomeles indica* is known to decrease NF-κB phosphorylation and expression of TNF-α, IL-1β, and IL-6 (Hu et al., 2022). *Chrysanthemum morifolium* suppresses NF-κB pathways, proinflammatory cytokines, and CRP levels (Li et al., 2019). *Glycyrrhiza glabra* regulates NF-κB to alleviate inflammation (Sun et al., 2021). *Houttuynia cordata* Thunb has been shown to reduce leukocytosis, lower proinflammatory cytokine levels, and downregulate the MAPK and NF-kB pathways (Das et al., 2022; Yang et al., 2022). *Ophiopogon japonicus* reduces activation of p65 and p-IκB and decreases pro-inflammatory cytokine expression (Mao et al., 2020). *Perilla frutescens* effectively inhibits inflammation by deactivating the HMGB1 signaling pathway and TNF-α and/or IL-6 (Yang et al., 2013; Wang et al., 2018). *Platycodon grandiflorus* inhibits the expression of NF-κB, TNF-α, IL-6, and caspase-3 (Tao et al., 2015). Based on these studies, the components of JSHT have demonstrated anti-inflammatory effects. In the current study, we further demonstrated the clinical benefits of JSHT in COPD, which include a reduction in symptoms such as cough and phlegm. Additionally, CRP levels were observed to decrease, indicating the anti-inflammatory effects of JSHT. Therefore, this study highlights both the clinical efficacy of JSHT in managing COPD and its anti-inflammatory properties at the cellular level.

A decrease in blood potassium levels was observed in the control group of COPDAE, but not in the JSHT group. The standard treatments for COPDAE, including intravenous steroids and inhaled beta-agonists are known to reduce potassium levels (Amegadzie et al., 2022; Wanas et al., 2025). Many botanical drugs are known to contain high levels of potassium. Specifically, botanical drugs such as *Artemisia argyi*, (Chen et al., 2022), *Anisomeles indica*, (Nasrin et al., 2022), *Glycyrrhiza glabra*, (Nasrin et al., 2022), *Houttuynia cordata* Thunb, (Luo et al., 2022), *Ophiopogon japonicus*, (Wang et al., 2017), and *Platycodon grandiflorus* (Ji et al., 2020) are rich in potassium; all these are included in the JSHT formulation (Hsieh et al., 2022a). When JSHT is used as an adjuvant therapy in combination with intravenous steroids and inhaled beta-agonists, it helps maintain stable potassium levels throughout the treatment course.

In recent years, several botanical drugs have been explored as adjunctive treatments in COPD due to their anti-inflammatory and immunomodulatory properties. In a randomized, double-blind, placebo-controlled trial, ginseng capsules did not significantly reduce the rate of acute exacerbations in patients with moderate to very severe COPD over 12 months, although it was safe and well tolerated (Chen et al., 2020). Secondary outcomes, including quality of life and lung function, showed minimal improvements (Chen et al., 2020). One systematic review of nine preclinical studies suggests that curcumin may have beneficial effects in managing COPD by reducing inflammation, oxidative stress, and airway remodeling (Safari et al., 2023). However, further randomized clinical trials are needed to confirm its efficacy in clinical settings (Safari et al., 2023). Casticin protects against COPD in cigarette smoke-exposed rats by improving lung function and reducing oxidative stress and inflammation through inhibition of the NF-κB and iNOS pathways (Li et al., 2020). Compared to these agents, JSHT demonstrated a robust suppression of IL-6 and TNF-α, as well as upstream mediators such as HMGB1, FPR1, and downstream signaling molecules including pMAPK, pJNK, and NF-κB in LPS-stimulated A549 cells. This broad-spectrum inhibitory profile suggests that JSHT may offer comparable or potentially therapeutic efficacy due to its multi-component formulation, which may act synergistically on several inflammatory pathways relevant to COPD pathogenesis. These findings support JSHT as a promising candidate for further clinical evaluation in the context of COPD adjunctive therapy.

In the current study, we used LPS stimulation in A549 cells as a cellular model of COPD. Bronchial epithelial cell line 2B and Murine Lung Epithelial-12 cell line are also commonly used cell lines for COPD research as they more closely resemble normal bronchial epithelial cells (Zhao et al., 2024; Xie and Wang, 2025). A549 cells, a well-established human lung carcinoma cell line derived from the alveolar epithelial cells of a Caucasian male, are also widely used in COPD-related studies (Sai et al., 2024; Jang et al., 2025). A549 cells provide valuable insights into how treatments modulate cellular responses to irritants and inflammation in COPD. Therefore, A549 cells remain a rational choice for this COPD study, particularly with a focus on inflammation and responses to treatments like JSHT.

**FIGURE 4:**
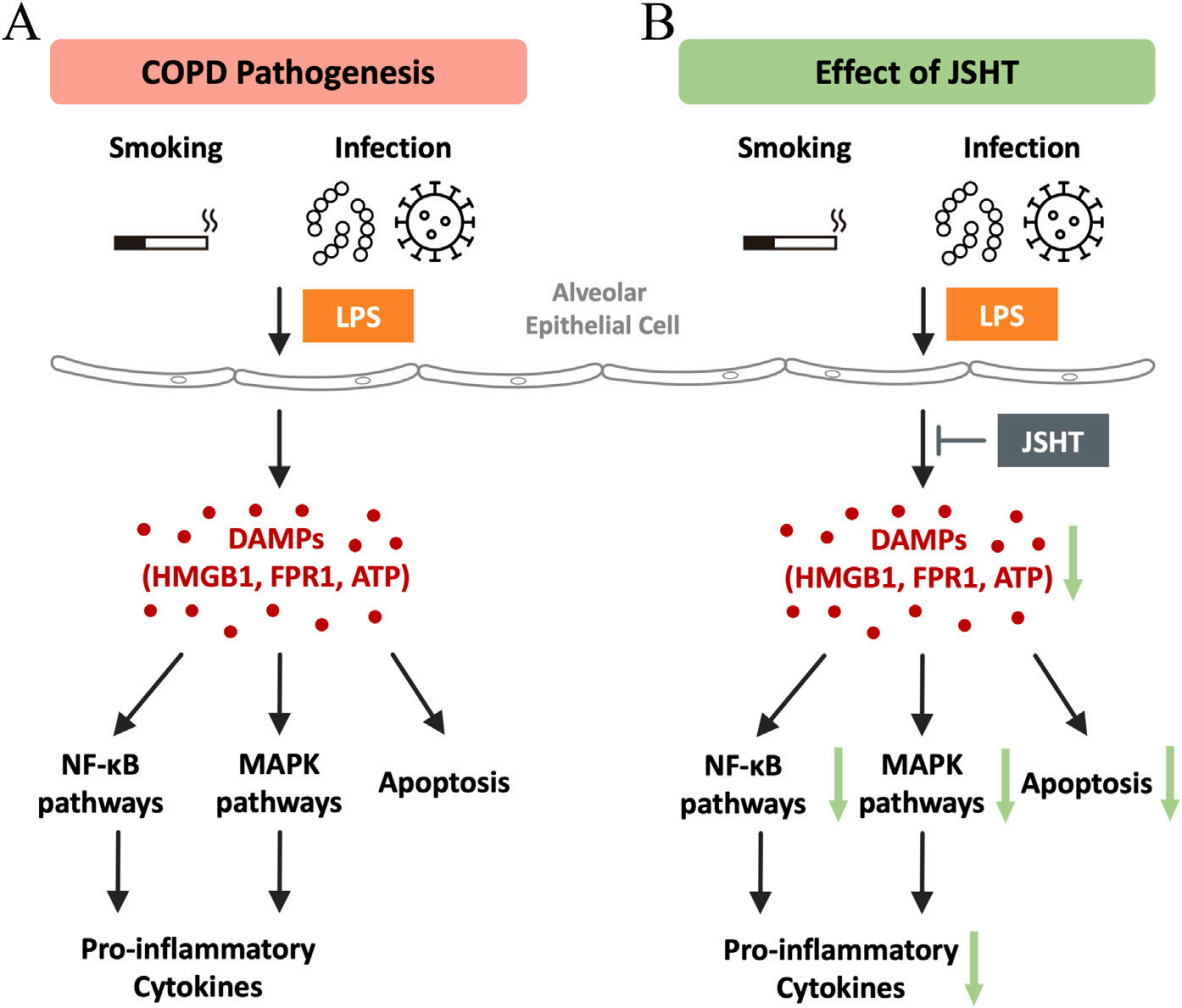
Mechanisms of the effects of JSHT in COPD The pathogenesis of COPD **(A)** involves the release of DAMPs by lung epithelial cells in response to injury, which triggers downstream inflammatory pathways like NF-κB, MAPK, and apoptosis, leading to increased pro-inflammatory cytokine production. Administration of JSHT **(B)** reduces DAMP levels, thereby attenuating NF-κB and MAPK pathways, apoptosis, and pro-inflammatory cytokines. Abbreviations: COPD: chronic obstructive pulmonary disease; JSHT: Jing Si Herbal Tea; DAMPs: damage-associated molecular patterns; HMGB1: high mobility group box 1; FPR1: formyl peptide receptor 1; ATP: adenosine triphosphate; NF-κB: nuclear factor kappa-light-chain-enhancer of activated B cells; MAPK: mitogen-activated protein kinases.

Figure 4 illustrates the pathogenesis of COPD and modulatory effects of JSHT. DAMPs are released by lung epithelial cells in response to injury, which is a critical event in the development of COPD. These DAMPs activate downstream inflammatory pathways, such as NF-κB, MAPK, and processes leading to apoptosis. This activation results in increased production of pro-inflammatory cytokines. Administration of JSHT effectively attenuates DAMPs and attenuates the activity of NF-κB and MAPK pathways, along with apoptosis and reduction in pro-inflammatory cytokines. These results demonstrate the potential of JSHT as a therapeutic agent for the management of COPD.

## 6 Clinical implication

JSHT has been approved by the Ministry of Health and Welfare of Taiwan (registration number MOHW-PM-060635) (Chiang et al., 2023). This is the first study to demonstrate the effects of JSHT on patients with COPD. The observed improvements in HRQL and symptom reduction during both the exacerbation and stable phases suggest that JSHT could complement standard COPD treatments. Cellular studies demonstrated JSHT’s anti-inflammatory effects by reducing the release of DAMPs and inflammatory markers.

### 6.1 Limitations of the study

This study had several limitations. First, the limited sample size in both the exacerbated and stable COPD groups may have reduced the statistical power of our analyses, potentially increasing the risk of Type II errors. As this study was designed as a preliminary investigation, the findings should be interpreted with caution. Larger, well-powered randomized controlled trials are warranted to confirm the therapeutic benefits of JSHT and to ensure the reproducibility and generalizability of the results. Despite the sample size limitations, this study offers meaningful preliminary insights into the potential benefits of JSHT as an adjunctive treatment for COPD. Second, the relatively short duration of treatment and follow-up limits our ability to assess the sustained effects and long-term safety of JSHT in patients with COPD. While the observed improvements in symptoms and inflammatory markers are encouraging, it remains unclear whether these benefits persist beyond the study period. An extended study duration with longer follow-up is essential to evaluate the durability of JSHT’s therapeutic effects, its impact on disease progression, exacerbation frequency, and long-term clinical outcomes. Third, this study is the lack of detailed chemical profiling of the JSHT formulation. As our primary focus was to evaluate the overall clinical efficacy and anti-inflammatory effects of JSHT in COPD, we did not conduct component-level analysis in this phase. To address this, future studies will incorporate high-performance liquid chromatography, mass spectrometry, and related phytochemical techniques to establish a chemical fingerprint of JSHT. These efforts will help elucidate the roles of individual constituents and support mechanistic validation at the molecular level. Fourth, this study lacks a direct assessment of oxidative stress, a key contributor to COPD pathogenesis. Cigarette smoke and related irritants induce excessive production of reactive oxygen species (ROS), contributing to epithelial injury, chronic inflammation, and disease progression. While our study focused on evaluating the anti-inflammatory effects of JSHT through the measurement of DAMPs and cytokine expression, we did not include assays to quantify ROS levels or antioxidant responses. As oxidative stress and inflammation are closely interconnected in COPD, future studies assessing the potential antioxidant properties of JSHT would provide a more comprehensive understanding of its therapeutic effects.

## 7 Conclusions

JSHT showed significant improvements in HRQL for patients with COPD, regardless of whether they were in a stable condition or had experienced exacerbations. JSHT led to improvements in symptoms such as cough, phlegm, chest tightness, breathlessness, limitation of activities, confidence in leaving home, sleep difficulties, and anxiety among patients with COPDAE. For patients with stable COPD, JSHT improved cough, phlegm, and dyspnea. Cellular models demonstrated a reduction in DAMPs and inflammation, suggesting the potential of JSHT as a therapeutic agent for COPD by attenuating inflammatory responses.

## Data Availability

The original contributions presented in the study are included in the article/supplementary material, further inquiries can be directed to the corresponding author.

## Abbreviations

ALT, alanine aminotransferase; AST, aspartate aminotransferase; ATP, adenosine triphosphate; BSRS-5, Brief Symptom Rating Scale-5; BUN, blood urea nitrogen; CAT, COPD assessment test; cCaspase-3, cleaved caspase-3; COPD, chronic obstructive pulmonary disease; Cr, creatinine; CRP, C-reactive Protein; DAMPs, damage-associated molecular patterns; FPR1, formyl peptide receptor 1; Hb, hemoglobin; Hct, hematocrit; HMGB1, high mobility group box 1; HRQL, health-related quality of life; IL, interleukin; JSHT, Jing Si Herbal Tea; LPS, lipopolysaccharide; mMRC, modified Medical Research Council; NF-κB, nuclear factor kappa-light-chain-enhancer of activated B cells; p-JNK, phosphorylated c-Jun N-terminal kinase; p-MAPK, phosphorylated mitogen-activated protein kinase; PLT, platelets; pPro-BNP, pro-brain natriuretic peptide; PFT, pulmonary function tests; TNF-α, tumor necrosis factor-alpha; UA, uric acid; WBCs, white blood cells.
